# *Saccharomyces cerevisiae* var. *boulardii* (HO12) Improves Loperamide-Induced Constipation in Rats *via* Modulation of Intestinal Neurotransmitters and SCFA Production

**DOI:** 10.4014/jmb.2508.08040

**Published:** 2025-12-09

**Authors:** Sekyung Lee, Sang Min Kim, Hyung Joo Suh, Sung Hee Han, Yejin Ahn

**Affiliations:** 1 Department of Integrated Biomedical and Life Science, Graduate School, Korea University, Seoul 02841, Republic of Korea; 2 L-HOPE Program for Learning Healthcare Optimizing Place-Based Equity, Korea University, Seoul 02841, Republic of Korea; 3 Institute of Human Behavior & Genetics, Korea University, Seoul 02841, Republic of Korea; 4 Korean Food Research Institute, Wanju-gun, Jeollabuk-do 55365, Republic of Korea

**Keywords:** Constipation, *Saccharomyces cerevisiae* var. *boulardii*, serotonin, gastrointestinal motility

## Abstract

Chronic constipation is a common gastrointestinal disorder characterized by impaired intestinal motility and dysbiosis of the gut microbiota. This study evaluated the therapeutic potential of the probiotic yeast *Saccharomyces cerevisiae* var. *boulardii* (HO12) on loperamide-induced constipation in rats. We assessed fecal parameters, intestinal transit, histological alterations, neurotransmitter levels, and short-chain fatty acid (SCFA) production. HO12 administration effectively alleviated constipation symptoms in a dose-dependent manner. It significantly increased fecal number, weight, water content, and improved gastrointestinal transit, indicating enhanced intestinal motility. HO12 also restored serotonin levels and the expression of serotonin-related genes, key regulators of enteric neurotransmission. Furthermore, HO12 increased the production of acetic acid and total SCFAs, contributing to a favorable intestinal environment. Histological analysis demonstrated that HO12 restored mucosal thickness, crypt cell distribution, and interstitial cells of Cajal, thereby supporting intestinal functional recovery. In conclusion, our results demonstrate that HO12 alleviates loperamide-induced constipation by modulating intestinal neurotransmitters, enhancing intestinal motility, and increasing SCFA production. These findings suggest that *S. boulardii* HO12 has strong potential as a therapeutic probiotic for constipation management.

## Introduction

Living environments such as increased stress, irregular and unbalanced diet, and lack of exercise are known to increase gastrointestinal (GI) diseases such as intestinal dysfunction, chronic constipation, irritable bowel syndrome, diarrhea, and enteritis [1]. Constipation is a common gastrointestinal disorder, affecting 12–17% of people, especially women and the elderly [2]. Constipation may increase intestinal reabsorption of fecal toxins, which has been associated with skin problems such as acne, blemishes, and aging [3]. In addition, there is a possibility that harmful substances produced by abnormal fermentation in the intestine due to constipation can cause headache, loss of appetite, abdominal discomfort, allergies, and further cause secondary diseases such as hemorrhoids, colonic diverticulosis, colorectal cancer, and cardiovascular disease [4].

Constipation can be alleviated by fiber intake, but fiber alone is often insufficient [5]. Conventional management typically combines dietary fiber supplementation with pharmacological agents, including bulking-forming, osmotic, and stimulant laxatives [6]. These drugs may be accompanied by abdominal distension and diarrhea, and in the case of stimulant laxatives, long-term administration may cause various complications [7]. In addition, prolonged use can lead to electrolyte imbalance and other systemic side effects, highlighting the need for safer alternatives.

Recently, as many studies on the effect of relieving constipation using probiotics have been published, interest in probiotics is increasing [8]. It was reported that intestinal microbiota abnormalities in constipation patients improved after taking probiotics [9]. The intestinal microbiota of constipation patients and healthy people show different trends [10, 11]. Probiotics produce short-chain fatty acids (SCFAs), which lower gut pH, modulate the gut microbiota, enhance intestinal peristalsis, and consequently reduce intestinal transit time [12, 13]. However, the beneficial effects of lactic acid bacteria (LAB) are not always consistent, and their efficacy may vary depending on the host’s microbiota composition, thereby highlighting the need to explore alternative probiotic strains.

The most representative microorganisms among probiotics are LAB. In addition to LAB, certain yeasts are also used in fermentation processes and are recognized for their health-promoting effects. Unlike LAB, which have long been used as probiotics, yeasts exhibit antibiotic resistance, suggesting their potential for broader applications. Among them, *Saccharomyces cerevisiae* var *boulardii* is the most popular human-derived yeast probiotic. *S. boulardii* has excellent acid and heat resistance, enabling its survival in the host intestine [14]. It can colonize the intestinal mucosa, modulate gut microbiota, strengthen the intestinal barrier, and regulate immune responses [15, 16]. This yeast has been applied therapeutically for chronic disorders, including bacterial diarrhea [17] and inflammatory bowel disease [18]. However, the widespread use of antibacterial agents in treating intestinal infections has accelerated the emergence of multidrug-resistant organism [19]. Yeast probiotics have therefore been proposed as an alternative approach to address this challenge.

Despite its established efficacy in diarrhea-related disorders, the role of *S. boulardii* in constipation has been insufficiently investigated. Therefore, to assess the potential of the yeast strain HO12 as a probiotic for constipation management, we examined its effects in a rat model of loperamide-induced constipation. Specifically, we investigated its effects on stool parameters, intestinal transit, and related physiological indices to establish its therapeutic potential as a probiotic for constipation treatment.

## Materials and Methods

### Experimental Design

Male Sprague–Dawley (SD) rats (6 weeks old) were obtained from Oriental Bio (Republic of Korea) for this study. After a 5-day acclimation period, a total of 40 rats were randomly divided into five experimental groups (*n* = 8 per group): a non-constipated normal group (NOR), a negative control group with induced constipation (CON), a positive control group receiving phenolphthalein (PC, 70 mg/kg), and two groups treated with a low or high dose of HO12 (YL: 1 × 10^9^ CFU/day, YH: 1 × 10^10^ CFU/day). Constipation was induced in all groups except NOR by oral administration of loperamide (5 mg/kg, Sigma–Aldrich, USA) twice daily for 5 days. Phenolphthalein, a stimulant laxative, was purchased from Sigma-Aldrich and used as a positive control to compare the constipation-relieving efficacy of HO12 [20]. All animals were kept in a controlled environmental conditions (20–22°C, 50–55% humidity, and a 12-h light/dark cycle) with *ad libitum* access to food and water. All experimental procedures were approved by the Institutional Animal Care and Use Committee of Korea University (KUIACUC-2022-0022).

### Assessment of Constipation

The efficacy of HO12 treatment was evaluated by measuring key parameters indicators of constipation. Fecal parameters, including the number, wet weight, and water content of fecals, were measured at 2-day intervals during the loperamide induction phase and then weekly thereafter. Fecal water content was determined by calculating the weight difference between fresh and dried samples, following a 24-h drying period at 60°C. Intestinal transit rate was assessed using the established activated charcoal method. After 18-h fast, rats were orally administered a 5% activated charcoal solution. Thirty minutes later, the rats were euthanized, and the gastrointestinal tract was excised. The length of the intestine traversed by the charcoal was measured and expressed as a percentage of the total intestinal length [21].

### Histological Analysis

Mice were euthanized under CO_2_ anesthesia, and colon tissues were collected, fixed in 10% neutral buffered formalin, embedded in paraffin, and sectioned (5 μm). Hematoxylin and eosin (H&E) staining was performed to evaluate the thickness of the colorectal mucosal and muscular layers [22]. Furthermore, tissue sections were stained for acidic mucus using the Alcian Blue (pH 2.5) stain kit (#H-3501; Vector Laboratories, USA) to determine the distribution of crypt cells. The deparaffinized sections were hydrated and stained with Alcian Blue solution for 30 min, rinsed with tap water, and counterstained with Nuclear Fast Red solution for 5 min. For immunohistochemical (IHC) analysis, tissue sections were subjected to antigen retrieval by heating at 98°C for 20 min and blocking endogenous peroxidase activity with 3% hydrogen peroxide. To visualize the distribution of interstitial cells of Cajal (ICCs) in colonic tissues, sections were incubated with anti-c-KIT antibody (sc-365504; Santa Cruz Biotechnology, USA). Stained sections were observed under a light microscope, and the positively stained areas were quantified using MATLAB software (MathWorks, USA).

### Analysis of Serotonin Levels

Serotonin levels in colon tissue were quantified using commercial ELISA kits (MyBioSource, USA). Briefly, 100 mg of tissue was homogenized in 1 mL of phosphate-buffered saline, and the homogenates were centrifuged at 10,000 ×*g* for 15 min at 4°C to obtain the supernatant. ELISA assays was then performed on the supernatants according to the manufacturer's instructions.

### Quantitative Real-Time PCR (qRT-PCR)

Total RNA was extracted from colon tissue (100 mg) using TRI reagent (Sigma–Aldrich). cDNA was synthesized from the isolated RNA using Oligo(dT) primers with SuperScript III reverse transcriptase (Thermo Fisher Scientific, USA). Quantitative PCR was performed on a real-time PCR system with SYBR Green Master Mix. Expression levels of target genes were normalized to the housekeeping gene (*GAPDH*) and calculated relative to the normal group (NOR).

### Western Blot Analysis

Colon tissue samples (100 mg) were homogenized in ice-cold RIPA buffer (Thermo Fisher Scientific) and centrifuged at 12,000 ×*g* for 5 min at 4°C to extract total protein. Protein concentrations were determined using a BCA assay. Equal amounts of protein (50 μg) were separated on 10% SDS-PAGE and transferred onto PVDF membranes. The membranes were blocked with 5% bovine serum albumin and incubated overnight at 4°C with the appropriate primary antibodies. Subsequently, they were incubated with HRP-conjugated secondary antibodies. Protein bands were detected using SuperSignal Western Blot Enhancer (Thermo Fisher Scientific) and imaged with a FluorChem M system (ProteinSimple, USA). The primary antibodies used were GAPDH (#5714), *ZO-1* (#sc-33725), C-kit (#ab256345), *MUC4* (#ab194363), and *AQP3* (#ab125219).

### SCFA Analysis

SCFA levels in cecal contents were determined using gas chromatography (GC 7890; Agilent Technologies, USA), with 2-Ethylbutyric acid was used as an internal standard [23]. The system was equipped with a flame ionization detector, an autosampler, and a DB-FFAP column (50 m × 0.32 mm × 0.50 μm). Cecal contents (100 mg) were extracted with 0.8 ml of 80% methanol, and the fecal extract was filtered through a 0.45 μm membrane and analyzed. The oven temperature program was as follows: initial temperature of 100°C was increased to 180°C at a rate of 8°C/min, held for 1 min, followed by increased to 200°C at a rate of 20°C/min and held for 5 min.

### Cecal Microbiome Analysis

Genomic DNA was extracted from mouse cecal contents using the QIAamp PowerFecal Pro DNA Kit (QIAGEN, USA). The composition of the cecal microbiota was analyzed by 16S rRNA gene sequencing using the Illumina MiSeq platform (Macrogen Inc., Republic of Korea) as previously described [24]. Alpha diversity was assessed using the Chao1, Shannon, and Gini-Simpson indices, and the relative taxonomic abundance was determined at the phylum and genus levels.

### Statistical Analysis

All statistical analyses were conducted using SPSS software (version 12.0; SPSS Inc., USA). Data are presented as mean ± standard error of the mean (SEM). Group differences were analyzed by one-way ANOVA with Tukey’s post hoc test.

## Results

### Fecal Parameters and Gastrointestinal Transit

**Fig. 1:**
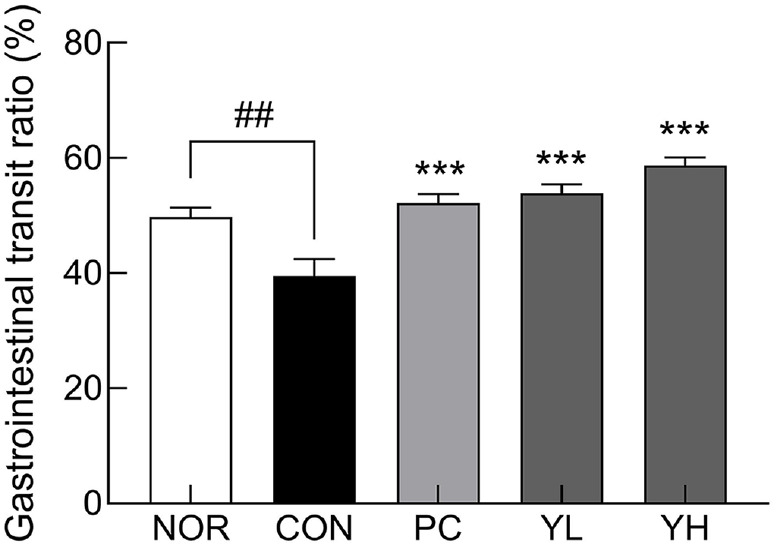
Effect of *Saccharomyces cerevisiae* var. *boulardii* HO12 on gastrointestinal transit ratio in loperamideinduced constipated SD rats. Data are expressed as the mean ± standard error of the mean (SEM) (n = 8). NOR: normal group, CON: loperamide-control group, PC: phenolphthalein (70 mg/kg); YL: HO12 low dose (1 × 10^9^ CFU/day), YH: HO12 high dose (1 × 10^10^ CFU/day). ^###^*p* < 0.001 vs. NOR, and ***p < 0.001 vs. CON by Tukey’s test.

The effects of HO12 administration on constipation were evaluated by analyzing fecal parameters (Fig. S1) and intestinal motility (Fig. 1). The CON group, which received loperamide alone, showed significant reductions in fecal number (*p* < 0.01), weight (*p* < 0.05), and water content (*p* < 0.001) compared to the NOR group, confirming the successful induction of constipation (Fig. S1). In contrast, HO12 administration significantly improved all fecal parameters in a dose-dependent manner compared to the CON group (*p* < 0.05, *p* < 0.01, and *p* < 0.001, respectively). Consistent with the fecal findings, intestinal motility was restored by HO12 treatment (Fig. 1). The GI transit rate in the CON group (39.5%) was significantly lower than in the NOR group (49.8%). However, HO12 administration significantly increased the GI transit ratio in a dose-dependent manner (YL: 53.9%, *p* < 0.001; YH: 58.7%, *p* < 0.001), which was higher than that in the PC group (52.2%, *p* < 0.001).

### Histopathology and Immunohistochemistry

**Fig. 2:**
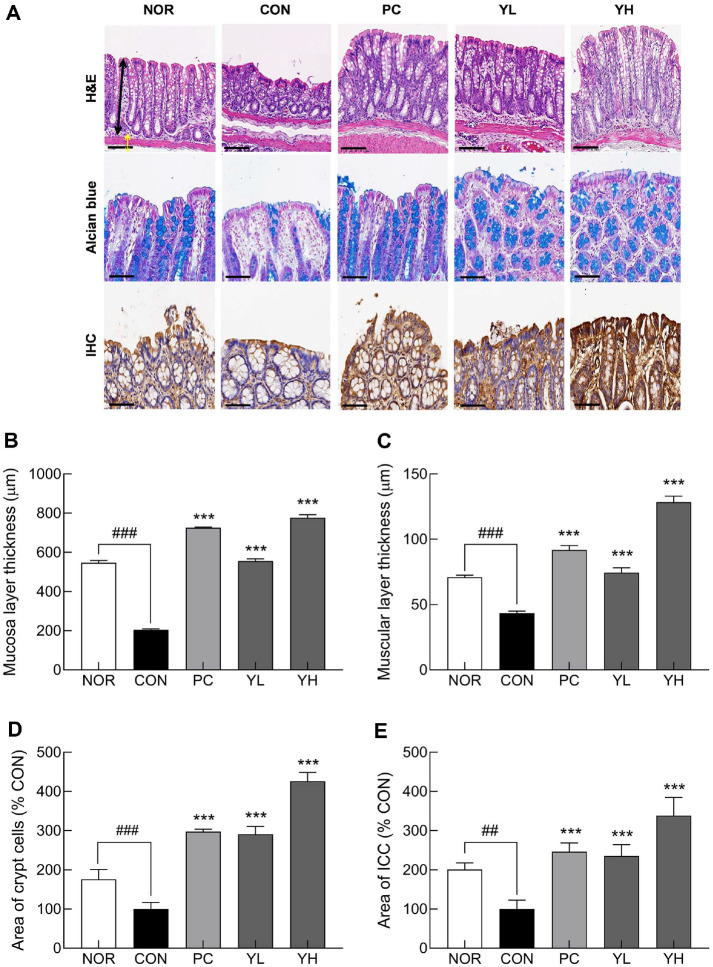
Effects of *S. cerevisiae* var. *boulardii* HO12 on histological changes in colonic tissues of loperamideinduced constipated SD rats. (**A**) Representative images of H&E, Alcian blue, and IHC staining of colonic tissues. Scale bars indicate 100 μm for H&E and 60 μm for Alcian blue and IHC; Thickness of (**B**) mucosal and (**C**) muscular layers; (**D**) area of goblet cells; and (**E**) area of interstitial cells of Cajal (ICC). Data are expressed as the mean ± standard error of the mean (SEM) (n = 8). NOR, normal group; CON, loperamide-treated control; PC, phenolphthalein (70 mg/kg); YL, HO12 low dose (1 × 10^9^ CFU/day); YH, HO12 high dose (1 × 10^10^ CFU/day). ^##^*p* < 0.01, ^###^*p* < 0.001 vs. NOR; ***p < 0.001 vs. CON by Tukey’s test.

Histological analysis of colonic tissues (Fig. 2A) revealed that the CON group exhibited significantly thinner mucosal layers (Fig. 2B; *p* < 0.001) and muscular layers (Fig. 2C; *p* < 0.001) compared to the NOR group (*p* < 0.001). However, HO12 administration dose-dependently improved mucosal (*p* < 0.001) and muscular layer (*p* < 0.001) thickness. In addition, the distribution of crypt cells, which are involved in mucus secretion, was significantly reduced in the CON group compared to the NOR group (Fig. 2D; *p* < 0.001), whereas HO12 treatment markedly increased the distribution of crypt cells (YL: *p* < 0.001; YH: *p* < 0.001) relative to the CON group. The distribution of ICCs, which regulate gastrointestinal peristalsis, was analyzed by IHC staining. Four weeks of loperamide administration significantly reduced the area of ICCs compared to the NOR group (Fig. 2E; *p* < 0.01). However, HO12 administration significantly restored the area of ICCs in a dose-dependent manner compared to the CON group. Notably, high-dose HO12 administration effectively improved the histological changes in colonic tissue induced by loperamide compared to the PC group.

### Serotonin Metabolism

**Fig. 3:**
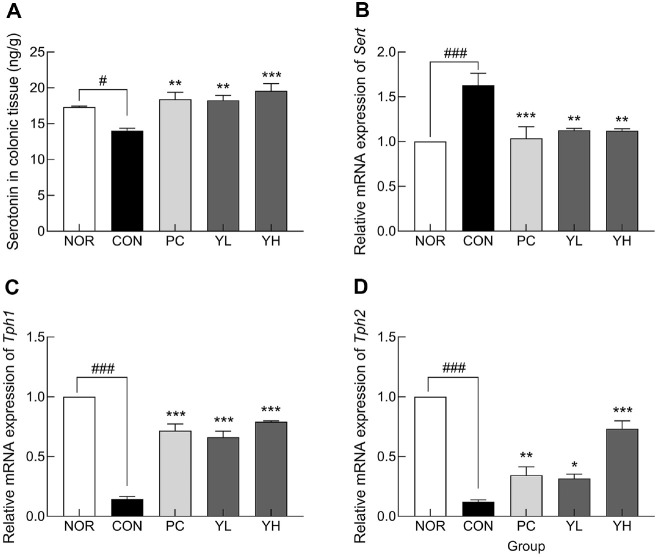
Effects of *S. cerevisiae* var. *boulardii* HO12 on serotonin content and serotonin metabolism-related mRNA expression in colonic tissues of loperamide-induced constipated SD rats. (A) serotonin content; relative mRNA expression of (B) *Sert*, (C) *Tph1*, and (D) *Tph2*. Data are expressed as the mean ± standard error of the mean (SEM) (*n* = 8). NOR: normal group; CON: loperamide-control group; PC: phenolphthalein (70 mg/kg); YL: HO12 low dose (1×10^9^ CFU/day); YH: HO12 high dose (1×10^10^ CFU/day). ^#^*p* < 0.05, ^###^*p* < 0.001 vs. NOR, and *p < 0.05, **p < 0.01, ***p < 0.001 vs. CON by Tukey’s test. Sert: serotonin transporter; Tph1: tryptophan hydroxylase-1; Tph2: tryptophan hydroxylase-2.

The effects of HO12 on serotonin regulation in the colonic tissue of loperamide-induced constipation rats are shown in Fig. 3. Serotonin levels in colonic tissue were significantly reduced in the CON group compared to the NOR group (Fig. 3A; *p* < 0.05). However, HO12 administration significantly increased serotonin content compared to the CON group, indicating the potential for restoration of serotonin-mediated intestinal motility (YL: *p* < 0.01; YH: *p* < 0.001). Analysis of serotonin-related gene expression revealed that the expression of *Sert*, involved in serotonin reuptake, was significantly increased in the CON group compared to the NOR group (Fig. 3B; *p* < 0.001). However, HO12 treatment significantly decreased *Sert* expression (*p* < 0.01). In contrast, the expression of *Tph1* and *Tph2*, which are involved in serotonin synthesis, was significantly downregulated in the NOR group compared to the CON group (Fig. 3C and 3D; *p* < 0.001), and this decrease was significantly reversed by HO12 administration. Notably, high-dose HO12 administration improved serotonin metabolism to levels similar to or higher than those in the PC group.

### Aquaporin and Tight Junction-Related Factors

**Fig. 4:**
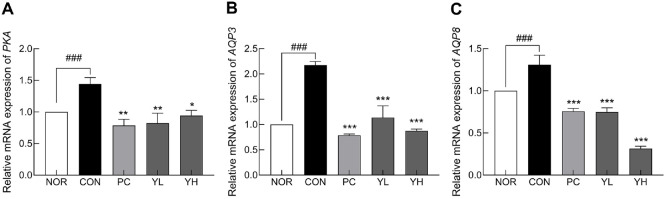
Effects of *S. cerevisiae* var. *boulardii* HO12 on the mRNA expression of (A) *PKA*, (B) *AQP3*, and (C) *AQP8* in the colonic tissues of loperamide-induced constipated SD rats. Data are expressed as the mean ± standard error of the mean (SEM) (n = 8). NOR, normal group; CON, loperamide-treated control; PC, phenolphthalein (70 mg/kg); YL, HO12 low dose (1 × 10^9^ CFU/day); YH, HO12 high dose (1 × 10^10^ CFU/day). ^###^*p* < 0.001 vs. NOR, and *p < 0.05, **p < 0.01, ***p < 0.001 vs. CON by Tukey’s test. *PKA*: protein kinase A; *AQP3*: aquaporin 3; *AQP8*: aquaporin 8.

Fig. 4 shows the effect of HO12 on the regulation of colonic water transport. Compared to the NOR group, the CON group significantly downregulated the mRNA expression of *PKA* (Fig. 4A; *p* < 0.001), *AQP3* (Fig. 4B; *p* < 0.001), and *AQP8* (Fig. 4C; *p* < 0.001). Conversely, HO12 treatment significantly upregulated aquaporin-related genes in a dose-dependent manner compared to the CON group (*PKA*: *p* < 0.01 and *p* < 0.05; *AQP3*: *p* < 0.001; *AQP8*: *p* < 0.001). High-dose HO12 restored *AQP3* and *AQP8* expression to levels comparable to or higher than those in the PC group.

**Fig. 5:**
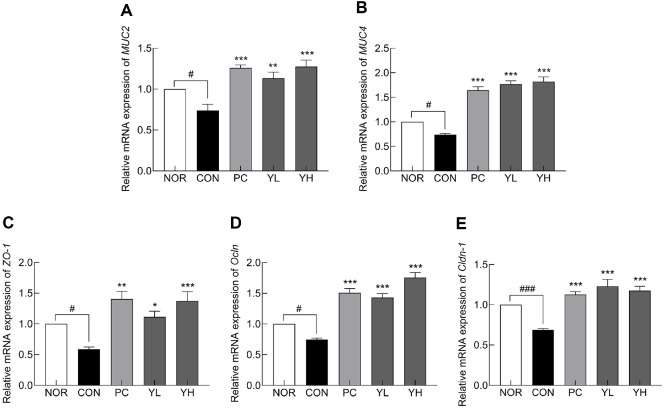
Effects of *S. cerevisiae* var. *boulardii* HO12 on mRNA expression of (A) *MUC2*, (B) *MUC4*, (C) ZO-1, (D) Ocln, and (E) *Cldn-1* in the colonic tissues of loperamide-induced constipated SD rats. Data are expressed as the mean ± standard error of the mean (SEM) (n = 8). NOR, normal group; CON, loperamide-treated control; PC, phenolphthalein (70 mg/kg); YL, HO12 low dose (1 × 10^9^ CFU/day); YH, HO12 high dose (1 × 10^10^ CFU/day). ^#^*p* < 0.05, ^###^*p* < 0.001 vs. NOR, and *p < 0.05, **p < 0.01, ***p < 0.001 vs. CON by Tukey’s test. *MUC2*: mucin 2, *MUC4*: mucin 4, *ZO-1*: zonula occludens-1, *Ocln*: occludin, *Cldn-1*: cludin-1.

**Fig. 6:**
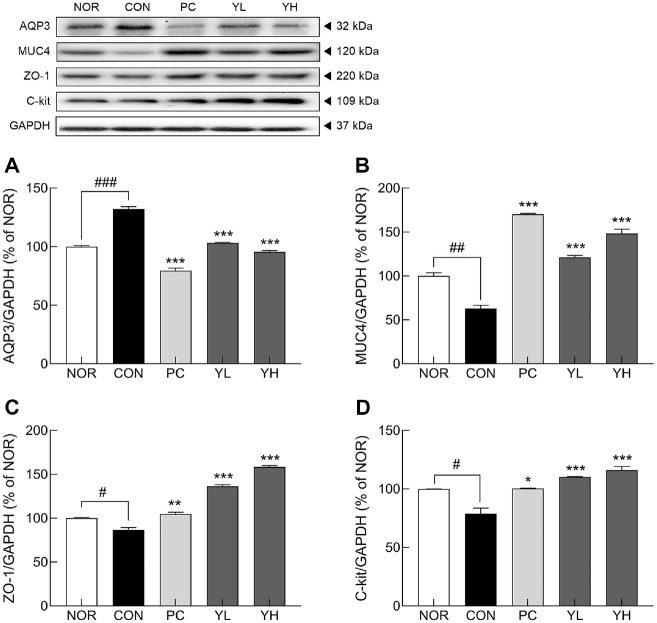
Effects of *S. cerevisiae* var. *boulardii* HO12 on protein level of (A) AQP3, (B) MUC4, (C) ZO-1, and (D) C-kit (D) in the colonic tissues of loperamide-induced constipated SD rats. Data are expressed as the mean ± standard error of the mean (SEM) (n = 8). NOR, normal group; CON, loperamide-treated control; PC, phenolphthalein (70 mg/kg); YL, HO12 low dose (1 × 10^9^ CFU/day); YH, HO12 high dose (1 × 10^10^ CFU/day). ^#^*p* < 0.05, ^##^*p* < 0.01, and ^###^*p* < 0.001 vs. NOR, and *p < 0.05, **p < 0.01, and ***p < 0.001 vs. CON by Tukey’s test. *AQP3*: aquaporin 3, *MUC4*: mucin 4, *ZO-1*: zonula occludens-1, C-kit: receptor tyrosine kinase protein.

Loperamide-induced constipation markedly reduced the mRNA expression of mucin genes (Fig. 5A and 5B; *p* < 0.05), whereas HO12 administration significantly restored the decrease in *MUC2* (YL: *p* < 0.01, YH: *p* < 0.001) and *MUC4* (*p* < 0.001). Furthermore, loperamide treatment significantly reduced the mRNA expression of tight junction markers compared to the NOR group (Fig. 5C-5E; *p* < 0.05 and *p* < 0.001, respectively). In contrast, HO12 administration significantly increased the expression of *ZO-1* in a dose-dependent manner (YL: *p* < 0.05, YH: *p* < 0.001) and also significantly restored the expression of *Ocln* and *Cldn-1* (*p* < 0.001). Consistent with the mRNA expression results, protein levels of AQP3 (*p* < 0.001), MUC4 (*p* < 0.01), ZO-1 (*p* < 0.05), and C-kit (*p* < 0.05) were significantly reduced in the CON group compared to the NOR group (Fig. 6). HO12 treatment markedly reversed this downregulation in a dose-dependent manner (*p* < 0.001). These findings indicate that HO12 effectively ameliorates loperamide-induced impairment of water transport, mucosal integrity, and intestinal motility by regulating aquaporin and tight junction-associated pathways.

### SCFA Production and Changes in Gut Microbiota

**Fig. 7:**
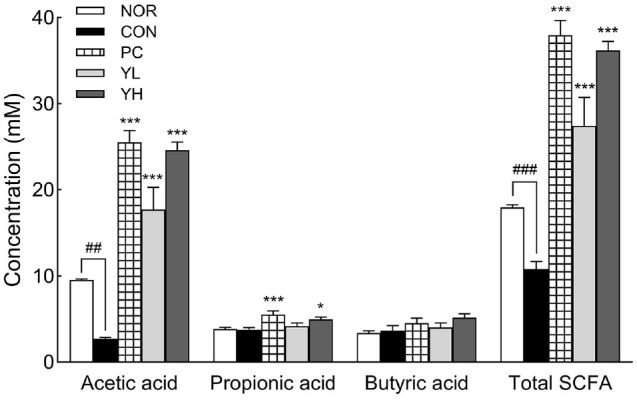
Effects of *S. cerevisiae* var. *boulardii* HO12 on SCFA production in the cecal contents of loperamideinduced constipated SD rats. Concentrations of acetic acid, propionic acid, butyric acid, and total SCFAs were determined by gas chromatography. Data are expressed as the mean ± standard error of the mean (SEM) (n = 8). NOR, normal group; CON, loperamide-treated control; PC, phenolphthalein (70 mg/kg); YL, HO12 low dose (1 × 10^9^ CFU/day); YH, HO12 high dose (1 × 10^10^ CFU/day). ^##^*p* < 0.01, ^###^*p* < 0.001 vs. NOR, and *p < 0.05, ***p < 0.001 vs. CON by Tukey’s test. SCFA: short chain fatty acid.

Caecal SCFA levels were significantly altered by loperamide-induced constipation (Fig. 7). Both acetic acid (*p* < 0.01) and total SCFA content (*p* < 0.001) were significantly reduced in the CON group compared to the NOR group. HO12 administration significantly increased acetic acid (*p* < 0.001) and total SCFA (*p* < 0.001) content compared to the CON group. In particular, propionic acid levels in the YH group significantly increased, and total SCFA levels returned to the levels of the PC group.

To further investigate the relationship between SCFA production and microbial composition, we performed 16S rRNA sequencing of the cecal microbiota. The α-diversity index tended to decrease in the CON group compared to the ND group, and the Gini-Simpson index was significantly decreased in the CON group (*p* < 0.05; Fig. S2). Conversely, the Shannon (*p* < 0.05) and Gini-Simpson (*p* < 0.001) indices were significantly higher in the YH group than in the CON group. At the phylum level, the CON group showed a tendency for the relative abundance of *Bacteroides*, *Actinobacteria*, and *Proteobacteria* to decrease compared to the NOR group. Conversely, the relative abundance of *Verrucomicrobia* increased (Fig. S3). However, high-dose HO12 administration significantly increased the relative abundance of *Firmicutes* (*p* < 0.05) and *Actinobacteria* (*p* < 0.05), while significantly decreasing the abundance of *Verrucomicrobia* (*p* < 0.05).

**Fig. 8:**
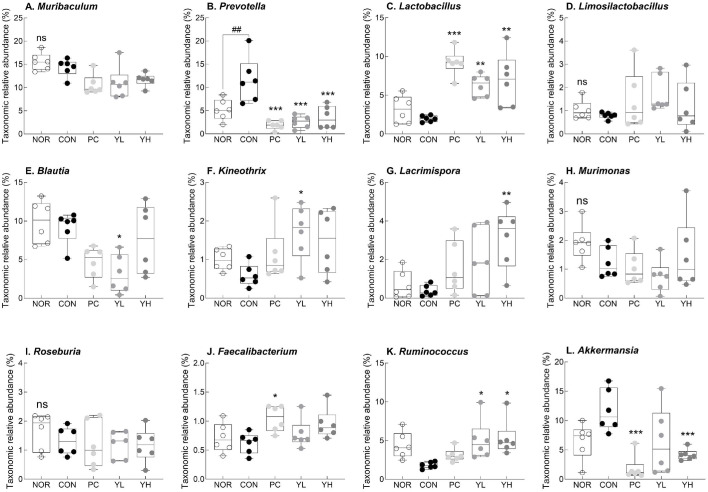
Effects of *S. cerevisiae* var. *boulardii* HO12 on gut microbiota composition at the genus level in loperamide-induced constipated SD rats. Relative taxonomic abundance of (**A**) *Muribaculum*; (**B**) *Prevotella*; (**C**) *Lactobacillus*; (**D**) *Limosilactobacillus*; (**E**) *Blautia*; (**F**) *Kineothrix*; (**G**) *Lacrimispora*; (**H**) *Murimonas*; (**I**) *Roseburia*; (**J**) *Faecalibacterium*; (**K**) *Ruminococcus*; and (**L**) *Akkermansia*. Data are expressed as the mean ± standard error of the mean (SEM) (n = 6). NOR, normal group; CON, loperamide-treated control; PC, phenolphthalein (70 mg/kg); YL, HO12 low dose (1 × 10^9^ CFU/day); YH, HO12 high dose (1 × 10^10^ CFU/day). ^##^*p* < 0.01 vs. NOR; *p < 0.05, **p < 0.01, and ***p < 0.001 vs. CON by Tukey’s test. ns, not significant.

At the genus level, loperamide administration significantly altered the gut microbiota composition compared to the NOR group (Fig. 8). In the CON group, the relative abundance of *Prevotella* significantly increased (*p* < 0.01; Fig. 8B), while the abundance of *Lactobacillus* (Fig. 8C), *Limosilactobacillus* (Fig. 8D), *Lacrimispora* (Fig. 8G), and *Ruminococcus* (Fig. 8K) tended to decrease. In contrast, HO12 administration significantly decreased the abundance of *Prevotella* (*p* < 0.001) in a dose-dependent manner, and the YH group showed a significant decrease in the abundance of *Akkermansia*. Furthermore, the relative abundance of *Lactobacillus* (*p* < 0.01) and *Ruminococcus* (*p* < 0.05) significantly increased in the HO12 group.

## Discussion

The potential health-promoting effects of probiotics have been elucidated by numerous studies. Recently, in addition to LAB, which is a representative probiotic, yeast such as *S. boulardii* has been used as a functional food material. However, studies on the use of *S. boulardii* as a probiotic are limited. Therefore, in this study, the constipation relief effect of *S. boulardii* (HO12) administration was evaluated in loperamide-induced SD rats. Loperamide is commonly used to induce constipation in experimental animals because it reduces colonic water and mucus secretion, inhibits colonic peristalsis, affects fecal excretion time, and delays intestinal transit [25, 26]. In this study, administration of loperamide also reduced the number, weight, and water content of feces and decreased intestinal mobility. HO12 administration was more effective than the stimulant laxative phenolphthalein in alleviating constipation symptoms (Fig. S1, Fig. 1). Previous studies have also reported that administration of probiotics modulates colonic transit time and alleviates functional GI disorders [27, 28].

Intestinal mucus is a lubricant in the digestive tract, which facilitates the movement of intestinal contents and protects the walls of the digestive tract [29]. HO12 administration contributed to the relief of constipation by restoring loss of crypt cells and ICCs caused by constipation (Fig. 2). The movement of the GI tract is caused by periodic contractions due to the spontaneous electrical activity caused by the slow waves generated in the ICC [30]. The ICC is electrically connected to the smooth muscle of the colon through a gap connection [31]. Constipation has been reported to decrease the number of ICCs in the smooth muscle of the colon and the number of neurons expressing excitatory neurotransmitters in the muscle layer [32].

Loperamide-induced constipation damages the intestinal mucosa, resulting in reduced expression of mucins (MUCs) and tight junction proteins (*e.g.*, ZO-1) in the intestinal epithelium [33]. *MUC2*, a major mucin expressed in colorectal cells, and *MUC4*, a membrane-bound mucin expressed in colon and small intestine cells, act as physical barriers [34, 35]. Mucins are the main component of the intestinal mucus layer, protecting epithelial cells from mechanical and chemical injury [36]. Tight junctions are protein complexes that regulate selective permeability and are composed of ZO-1, Ocln, and Claudins, among other proteins [37]. Reduced expression of tight junction molecules has been reported in patients with ulcerative colitis [38]. HO12 administration restored the mRNA and protein levels of mucins and tight junction markers that were reduced by loperamide, thereby contributing to the restoration of intestinal barrier integrity (Figs. 5 and 6). Similarly, administration of *L. plantarum* T34 has been reported to alleviate loperamide-induced constipation by restoring the thickness of the colonic muscle layer and upregulating the expression of claudin-1 and MUC3, thereby enhancing mucosal barrier function [39].

Serotonin, secreted by enteric nerve cells, is mainly distributed in the intestinal system and is known to promote the secretion of water and electrolytes, as well as to increase intestinal motility [40]. Approximately 95% of serotonin in the human body is present in the GI tract, synthesized from tryptophan by Tph1 in enterochromaffin (EC) cells [41]. Serotonin released by EC cells is absorbed into cells by SERT and then metabolized, thereby reducing the level of bioavailable serotonin in the intestine. Abnormal expression of SERT has been linked to GI dysfunction [42], and a decrease in serotonin has been reported in the colonic mucosa of patients with chronic constipation [43]. Gut microbes and their metabolites, SCFAs, contribute to the regulation of intestinal motility through serotonergic signaling [44]. SCFAs enhance serotonin biosynthesis in EC cells by stimulating Tph1 expression, and the increased serotonin activates enteric neurons and promotes peristalsis, thereby improving intestinal transit [45]. In this study, HO12 administration not only increased SCFA production (Fig. 7) but also improved serotonin levels by regulating the expression of serotonin synthesis-related genes (Fig. 3). These effects were accompanied by the restoration of aquaporin (Fig. 4) and tight junction markers expression (Fig. 5), leading to improved intestinal motility. Previous studies have shown that mice transplanted with fecal microbiota from patients with chronic constipation upregulated SERT levels in their intestinal tissue, reducing intestinal serotonin and impairing intestinal peristalsis [46]. Moreover, serotonin signaling has been reported to increase *AQP3* expression by acting as a ligand for peroxisome proliferator-activated receptor γ, thereby influencing colonic water transport [47]. Aquaporins are water channel proteins involved in fluid movement across the intestinal epithelium, with *AQP3* being abundantly expressed in the proximal colon, where it facilitates water reabsorption from the intestinal lumen into blood vessels. Activated PKA promotes stool hardening by increasing *AQP3* expression [48].

Accumulating evidence suggests that the gut microbiota plays a key role in the pathophysiology of constipation [9]. Alterations in the gut microbial community are closely associated with intestinal motility, mucus secretion, and water balance [49]. Patients with chronic constipation typically exhibit a decreased abundance of *Bifidobacterium* and *Lactobacillus*, along with an increased abundance of potentially pathogenic bacteria. Treatment with the stimulant laxative bisacodyl has been reported to normalize gut microbiota composition and improve intestinal function [50]. In the present study, HO12 administration increased microbial diversity (Fig. S2) and the abundance of *Lactobacillus* and *Limosilactobacillus* (Fig. 8). SCFAs produced by gut microbiota are involved in the differentiation and proliferation of colonocytes and reduce intestinal pH, inhibiting the growth of harmful microorganisms [51]. Among them, butyrate contributes to the integrity of the colonic epithelium and the regulation of inflammation. *Ruminococcus*, *Faecalibacterium*, and *Roseburia* are representative butyrate-producing genera [52]. Patients with slow-transit constipation had lower stool SCFA levels compared to healthy controls [53]. Consistent with these findings, HO12 administration was shown to alleviate the constipation-induced decrease in SCFA production by increasing the relative abundance of *Lacrimispora* and *Ruminococcus* (Fig. 8). Similary, administration of *Lactobacillus rhamnosus* LRa05 has been reported to relieve loperamide-induced constipation by modulating gut microbiota composition and increasing the abundance of SCFA-producing genera such as *Alloprevotella* and *Lachnospiraceae* [54]. Furthermore, *S. boulardii* treatment has been reported to improve the fecal microbiota composition and increase SCFA production in patients with chronic diarrhea [55]. *Akkermansia muciniphila* is generally regarded a beneficial mucus-degrading bacterium, its overgrowth can lead to excessive mucus degradation and compromise intestinal barrier [53]. Therefore, decreased abundance of *Akkermansia* observed in the HO12-treated group may reflect improved mucosal integrity (Fig. 8).

Collectively, administration of *S. cerevisiae* var. *boulardii* HO12 enhanced serotonin signaling and restored intestinal barrier integrity by modulating aquaporin expression. Furthermore, HO12 administration effectively alleviated loperamide-induced constipation by improving the composition and metabolic activity of the gut microbiota. These findings suggest that HO12 represents a promising probiotic candidate for the management of constipation and intestinal barrier dysfunction.

*S. boulardii* is a suitable strain for use in health supplement or functional food ingredient for promoting intestinal health due to its heat stability and gastric acid resistance [56, 57]. Clinically, *S. boulardii* has been widely used for the prevention and treatment of antibiotic-associated and infectious diarrhea, demonstrating safety and tolerability in both adults and children. Although *S. boulardii* is generally recognized as safe, rare cases of fungemia have been reported in severely immunocompromised or critically ill patients [17]. Therefore, future clinical applications of HO12 should include careful safety evaluation and dose optimization.

## Supplemental Materials

Supplementary data for this paper are available on-line only at http://jmb.or.kr.
