# Supplementary material for: Saccharomyces cerevisiae var. boulardii (HO12) Improves Loperamide-Induced Constipation in Rats via Modulation of Intestinal Neurotransmitters and SCFA Production
Source: J Microbiol Biotechnol. 2025 Dec 9;35:e2508040. doi: 10.4014/jmb.2508.08040 (PMC12711390; doi:10.4014/jmb.2508.08040)
Supplement: Supplementary file 1 [file jmb-35-e2508040-supple.pdf]

J. Microbiol. Biotechnol. 2025. 35: e2508040

https://doi.org/10.4014/jmb.2508.08040

Supplementary Figures

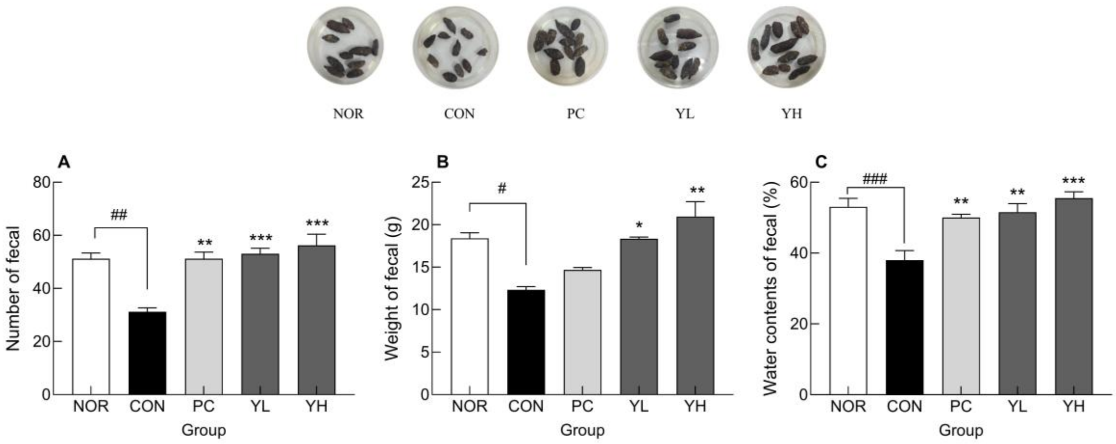

Fig. S1. Effects of *Saccharomyces cerevisiae* var. *boulardii* HO12 on fecal parameters in loperamide-induced constipated SD rats. (**A**) number, (**B**) weight, and (**C**) water content of fecal pellets. Data are expressed as the mean ± standard error of the mean (SEM) (n = 8). NOR: normal group; CON: loperamide-control group; PC: phenolphthalein (70 mg/kg); YL: HO12 low dose (1×10^{9} CFU/day); YH: HO12 high dose (1×10^{10} CFU/day). NOR: normal group, CON: loperamide-control group, PC: phenolphthalein (70 mg/kg); YL: HO12 low dose (1×10^{9} CFU/day), YH: HO12 high dose (1×10^{10} CFU/day). $^{\#}p$<0.05, $^{\#\#}p$<0.01 and $^{\#\#\#}p$<0.001 vs. NOR, and $^{*}p$<0.05, **p<0.01, $^{***}p$<0.001 vs. CON by Tukey's test.

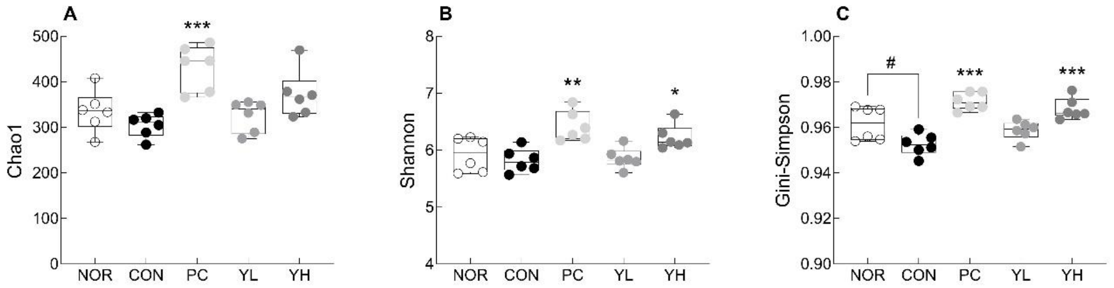

Fig. S2. Effects of *Saccharomyces cerevisiae* var. *boulardii* HO12 on alpha diversity indices in the cecal microbiota of loperamide-induced constipated SD rats. (A) Chao1 richness index; (B) Shannon diversity index; and (C) Gini-Simpson evenness index. Data are expressed as the mean ± standard error of the mean (SEM) (n = 8). NOR: normal group; CON: loperamide-control group; PC: phenolphthalein (70 mg/kg); YL: HO12 low dose (1×10^9 CFU/day); YH: HO12 high dose (1×10^{10} CFU/day). NOR: normal group, CON: loperamide-control group, PC: phenolphthalein (70 mg/kg); YL: HO12 low dose (1×10^9 CFU/day), YH: HO12 high dose (1×10^{10} CFU/day). $^{\#}p<0.05$ vs. NOR, and $^{*}p<0.05$, $^{**}p<0.01$, $^{***}p<0.001$ vs. CON by Tukey's test.

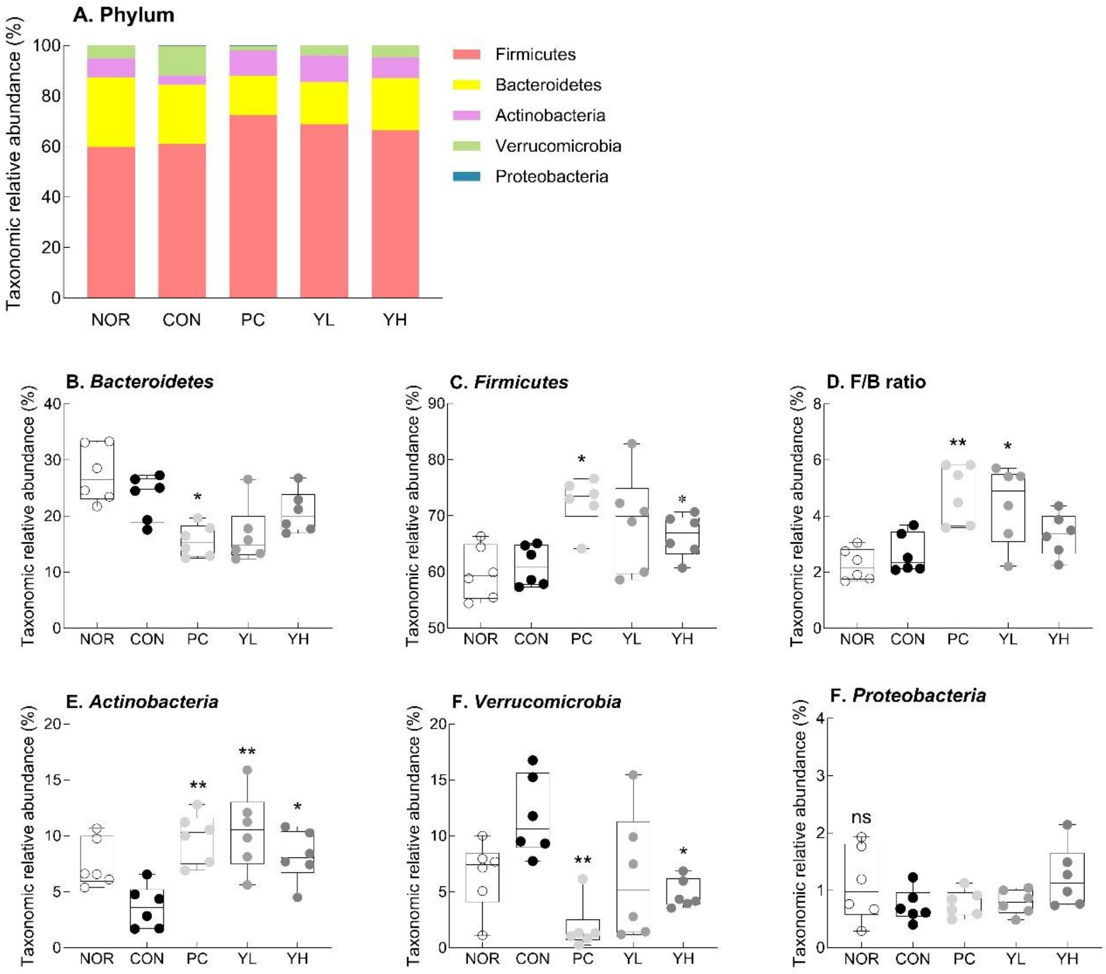

Fig. S3. Effects of *Saccharomyces cerevisiae* var. *boulardii* HO12 on cecal microbiota composition at the phylum level in loperamide-induced constipated SD rats. (A) Relative taxonomic abundance of major bacterial phyla; **(B)** *Bacteroidetes*; **(C)** *Firmicutes*; **(D)** *Firmicutes*/*Bacteroidetes* (F/B) ratio; **(E)** *Actinobacteria*; **(F)** *Verrucomicrobia*; and **(G)** *Proteobacteria*. Data are expressed as the mean ± standard error of the mean (SEM) (n = 6). NOR, normal group; CON, loperamide-treated control; PC, phenolphthalein (70 mg/kg); YL, HO12 low dose (1 × 10^9 CFU/day); YH, HO12 high dose (1 × 10^{10} CFU/day). *$p < 0.05$, **$p < 0.01$ vs. CON by Tukey's test. ns, not significant.
